# Experiences of imagery in obsessive‐compulsive disorder: An interpretative phenomenological analysis

**DOI:** 10.1111/bjc.12518

**Published:** 2024-12-05

**Authors:** Hannah E. F. Wedge, Louise Waddington, Andrew R. Thompson

**Affiliations:** ^1^ South Wales Doctoral Programme in Clinical Psychology, School of Psychology Cardiff University Cardiff UK; ^2^ Cardiff and Vale University Health Board Heath Park Cardiff UK; ^3^ Present address: Aneurin Bevan University Health Board, Headquarters St Cadoc's Hospital Newport UK

**Keywords:** interpretative phenomenological analysis, mental imagery, obsessive‐compulsive disorder, qualitative research

## Abstract

**Introduction:**

Mental imagery is a defining criterion within current OCD diagnoses, and yet little has been written about how this is experienced. This study aimed to investigate how people with a diagnosis of OCD experience imagery, to better understand how this might contribute to the condition.

**Methods:**

This research employed Interpretative Phenomenological Analysis (IPA) and used semi‐structured interviews. An expert‐by‐experience was involved in the study design. Eight adults with an OCD diagnosis were purposively sampled from NHS mental health services and interviewed about their experience of imagery. Interviews were transcribed and analysed in accordance with IPA guidelines. A reflexive log and audit trail were maintained during the research process to enhance quality control and to support the analytical process.

**Results:**

Six superordinate themes were found: *Submersion in intense and multifaceted imagery*; *Overwhelming, uncontrollable imagery*; *Imagery is explosive and expansive*; *Imagery involves past memories and future fears*; *People respond to imagery as if it is real*; *Therapy shifts imagery*.

**Conclusions:**

This study highlights the intensity of OCD‐related imagery experienced by people with OCD and the significance of this imagery in their everyday lives. All participants experienced imagery related to their OCD, demonstrating its importance in the phenomenology of the condition. Images relating to past experiences and images of future fears were identified. Clinicians should routinely seek to include imagery in assessment, formulation, and individual treatment plans for those with OCD.

**Practitioner points**

- People with OCD described experiencing imagery related to their condition as an intense and significant part of their everyday lives, forming a key part of their affective experience of living with OCD. This imagery was vivid, involving multiple sensory elements, and was often responded to as if it were depicting real‐life events, contributing to the compulsive element of the condition.
- OCD‐related imagery depicted not only future fears, but also negative past experiences, including those which were traumatic. More neutral memories related to people's obsessions and compulsions were also integrated into imagery, including things seen on television and in daily life.
- Clinicians should proactively engage with imagery as part of their work with people with OCD, including in assessment, as part of formulation, and woven into treatment plans; participants noted that therapy had been helpful in changing their experiences of imagery and the impact this has on them.

## INTRODUCTION

Mental imagery has been a phenomenon of research interest in psychological disorders for many years (e.g. Day et al., 2004; Hackmann et al., 1998), in addition to being studied as a normal cognitive function (Kosslyn et al., 2001) and one of the key manifestations of thoughts in our consciousness (Hackmann et al., 2011; Stopa, 2021). Holmes and Mathews (2010) define mental imagery as “neural representations constructed from more elemental sensory information” (p. 350) and add that “imagery can involve multiple sensory modalities, including bodily sensations and feelings” (p. 350). It is clear from these definitions that mental imagery is not simply “pictures in your mind's eye”, but is more complex and multisensory. Imagery has a strong relationship with emotion (Hackmann et al., 2011), including distress; as such, it is likely to be a feature of many existing categorizations of “psychological disorder”, from post‐traumatic stress disorder (PTSD) and anxiety disorders, to mood disorders and psychosis (Hackmann & Holmes, 2004).

The Diagnostic and Statistical Manual of Mental Disorders, 5th edition (DSM‐5; American Psychiatric Association (APA), 2013) includes recurrent and persistent images in the criteria for diagnosis of obsessive‐compulsive disorder (OCD); despite this, there has been limited research into imagery in OCD. De Silva (1986) used case studies to classify types of OCD imagery, but commented that there was little in the way of published research around OCD‐related imagery. Since then, other than a few case studies looking tangentially at the links between trauma, imagery, and OCD symptoms (De Silva & Marks, 1999; Lipinski & Pope, 1994), there have only been two studies which set out to explore imagery in OCD (Lipton et al., 2010; Speckens et al., 2007).

Speckens et al. (2007) used semi‐structured interviews and measures of OCD, depression, and anxiety to explore the prevalence, characteristics, and features of mental images in OCD. They studied a consecutive sample of patients admitted to an inpatient treatment programme for OCD, who had chronic, treatment‐resistant OCD, and may therefore not be fully representative of the wider clinical population of people with OCD. They found that 81% of their sample experienced imagery related to their OCD. Imagery was reported to be mostly experienced visually, though it also included all other sensory modalities. Participants who experienced imagery associated with OCD were found to have higher levels of symptomatology compared with people who did not have imagery. The authors found that the majority of images experienced as part of OCD were memories of adverse events, or images associated with these events.

Comparing a group of participants with OCD with a group of participants with non‐OCD anxiety disorders to ascertain whether imagery in OCD was distinguishable from imagery in other anxiety disorders, Lipton et al. (2010) found that there were no significant differences between these groups in imagery prevalence, vividness, or imagery‐related distress. The authors did however find that the frequency of intrusive imagery per week was significantly higher for people with OCD than for those with other anxiety disorders. Both groups reported primarily visual imagery, but more people in the OCD group than in the other anxiety disorders group reported that their visual imagery was from a field perspective (from their own point of view), as opposed to an observer perspective (from another's point of view). Contrary to the findings of Speckens et al. (2007), it was found that images in OCD were less likely to be associated with earlier memories than images in other anxiety disorders, but more likely to include future harm and to be drawn from imagination.

It is interesting that Speckens et al. (2007) found that 19% of their sample did not experience imagery; they acknowledged that this might have resulted from the way imagery was measured within the study. They used a binary measure of whether participants experienced mental imagery or thoughts, which may have meant that data was not captured for participants who described their experiences as a combination of thoughts and images. They suggested that it could be useful for future studies to use a more dimensional measure of the nature of intrusions. Lipton et al. (2010) suggested that future studies could also benefit from further investigating how participants distinguish the similarities and differences between images and memories, as there was scope for different interpretations in the way their own questions were phrased.

The aforementioned strong link between imagery and emotion (Hackmann et al., 2011) indicates that experiencing imagery is likely to be an important maintaining factor for psychopathology, especially given that imagery elicits more pronounced emotional responses than do verbal representations (Holmes & Mathews, 2010). People with diagnoses of psychological disorders, as well as chronic health conditions, can experience imagery as predictive, compelling, and holding important meaning (e.g. De Nicola et al., 2024; Hales et al., 2015), indicating that it may play a role in maintaining the distress associated with these difficulties. Imagery is also experienced “as if real”, to such an extent that fear‐related mental imagery has been shown to evoke similar physiological responses from the peripheral nervous system as if experiencing these images in “real life” (Ji et al., 2016). When considering the above in the context of the typical nature of obsessions and compulsions in OCD (which are often distressing and incongruent with the person's morals and values; Abramowitz, 2017), the importance of imagery as a potential maintaining factor for OCD becomes evident, and indicates that it is a phenomenon which is important to consider in our understanding of the disorder.

Investigating the nature of images in OCD is centrally important to understanding the experience of OCD and developing therapeutic interventions that cover all features of OCD. Should people experience images as being linked to adverse memories, therapeutic interventions similar to those used in PTSD treatment, such as imagery modification or imaginal reliving (Ehlers & Clark, 2000), may help to change the meanings and quality of memories associated with images. If images are experienced as being linked to future feared scenarios, therapeutic approaches, such as behavioural experiments, reducing cognitive avoidance, or rescripting (Arntz & Weertman, 1999) may be more helpful. Clearly, it is currently challenging to draw conclusions on this, as aside from the mixed‐methods studies by Speckens et al. (2007) and Lipton et al. (2010), there is little to be found on how people with OCD experience imagery, and no fully qualitative studies. There are also, to date, no studies of people's experiences of imagery in OCD using detailed phenomenological approaches. The use of such approaches as Interpretative Phenomenological Analysis (IPA: Larkin & Thompson, 2012; Smith et al., 2009), can be beneficial in this area, as IPA includes a focus on getting a close understanding of elements of specific experiences, making it a highly suitable approach to investigating mental imagery in OCD. IPA also takes a critical‐realist stance which emphasizes that it is possible to build a rich understanding of a specific set of individuals' experiences around a given phenomenon, and subsequently look at any patterns of similarity and difference across individual narratives (Eatough & Smith, 2017). The current study aims to build on the existing research to better understand the experience of OCD‐related mental imagery, using IPA to gain a rich insight into this phenomenon.

## METHODS

### Design

The study used Interpretative Phenomenological Analysis (IPA: Larkin & Thompson, 2012; Smith et al., 2009; Smith & Nizza, 2021), a qualitative approach which focuses on investigating people's experiences. IPA is idiographic, such that it focuses on the meaning of a given experience to a participant, and recognizes its significance for them, rather than attempting to fit them to pre‐defined theories (Larkin & Thompson, 2012). There is a double hermeneutic process in IPA, in which the participant is trying to make sense of their own experiences, and the researcher aims to make sense of that sense‐making (Smith, 2011). IPA is well suited to exploring people's experiences of illness, including mental ill‐health, as it gives voice to the experiences of participants, gives voice to the meaning of these experiences, and does not make initial assumptions (Biggerstaff & Thompson, 2008).

An expert‐by‐experience collaborated on this project on a paid consultancy basis. They were involved in developing and finalizing the interview schedule, as well as providing feedback on the aims and design of the study to ensure that the research is relevant and interesting to participants and the wider community of people with OCD; a significant benefit of involving experts‐by‐experience in qualitative research (Faulkner, 2012).

### Participants

Participants were eligible if they were over 18, had a diagnosis of OCD, and were experiencing imagery as part of this. Participants self‐identified as experiencing imagery related to their OCD, in keeping with the IPA approach, and no participants in the study experienced aphantasia. Current psychosis, drug/alcohol misuse, and hoarding as the primary or only aspect of OCD were exclusion criteria.

Participants were purposively sampled from a specialist NHS OCD service and Psychological Therapies service. None of the authors were working within these services, clinically or otherwise. In addition, the expert‐by‐experience was invited and agreed to participate in the study. The study was also advertised through OCD Action, a national OCD charity, however, no participants were recruited via this method. Eight participants were sought as this was deemed an appropriate sample size that still allows detailed analysis of individual experience to occur and is as such commensurate with the IPA approach (Smith, 2011; Smith et al., 2009). See Table 1 for participant characteristics.

**TABLE 1:** Participant characteristics.

Participant^a^	Age	Gender (pronouns^b^)	Ethnicity^b^	Time since OCD diagnosis	Comorbidities^b^	Treatment	PHQ‐9 depression severity	GAD‐7 anxiety severity	OCI score^c^	OCI focus
Dan	34	Male (he/him)	White British	1 year	Generalized Anxiety Disorder	Current: CBT group	Moderate	Severe	82	Checking
Hattie	19	Female (she/her)	White British	4 months	None	Current: CBT group	Severe	Severe	162	Washing, Checking, Obsessions
Rachel	27	Female (she/her)	White British	13 years	None	Previous: CBT group	Mild	Moderate	73	Obsessions
Amiera	26	Female (she/her)	British Pakistani	1 year	None	Previous: CBT group	Severe	Severe	80	Washing
Carys	32	Female (she/her)	White British	2 years	Depression	Current: CBT group	Moderately severe	Severe	47	Washing
Saskia	19	Female (she/her)	White	3 months	None	Current: Individual CBT	Moderate	Moderate	97	Washing, Checking
Natalie	21	Female (she/they)	White	6 months	Depression	Current: Group CBT	Severe	Severe	85	Obsessions
					Anxiety					
					Agoraphobia					
					ADHD Awaiting autism assessment					
Bridget	67	Female (she/her)	British	47 years	Childhood anorexia	Previous: “Flooding” treatment, CBT, CAT, EMDR	Moderately severe	Severe	146	Checking

*Note*: PHQ‐9: Patient Health Questionnaire‐9 (Kroenke et al., 2001); GAD‐7: Generalized Anxiety Disorder‐7 (Spitzer et al., 2006): OCI: Obsessive‐Compulsive Inventory (Foa et al., 1998).

Abbreviations: CAT, Cognitive Analytic Therapy; CBT, Cognitive Behavioural Therapy; EMDR, Eye Movement Desensitization and Reprocessing.

^a^ Pseudonyms to protect identities.

^b^ Self‐identified pronouns, ethnicity, and comorbidities.

^c^ OCI range: 0–168; clinical cut‐off score: 40 (Foa et al., 1998).

### Procedure

NHS ethical approval was granted prior to commencement of the study (IRAS Project ID 295975). The study was shared with potential participants by word of mouth and poster advertisements in therapy rooms. Therapists shared the information sheets with individual patients and the first author visited group sessions to give a short introduction to the research and answer any questions. Eligibility was confirmed by the first author via telephone or email and participants were subsequently contacted via email or telephone to arrange the interview. Interviews were held either in person in clinic rooms, or virtually (via a secure version of Microsoft Teams). All interviews were conducted by the first author. At the start of the interview, participants were asked to review the information sheet, invited to ask further questions, and asked to provide informed consent for the interview and sign the consent form. Interviews were recorded and transcribed verbatim. Interviews lasted between 41 and 77 min.

### Data collection

**FIGURE 1:**
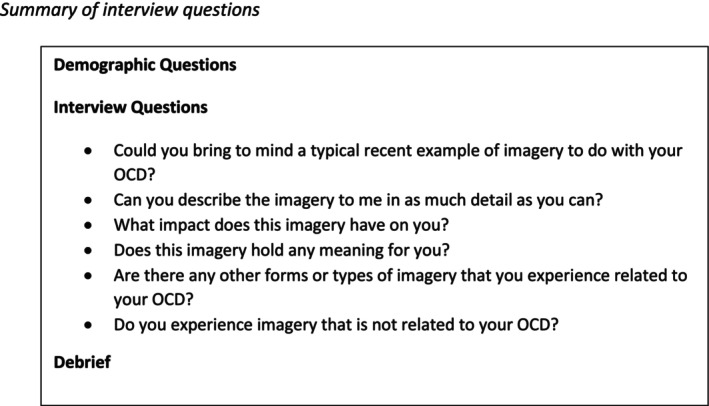
Summary of interview questions.

Demographic information was collected at the beginning of the interview (the full semi‐structured interview schedule is available in File S1). The interview schedule was developed with contributions from Consultant Clinical Psychologists with specific Cognitive Behavioural Therapy expertise and experience working with people with OCD, and the expert‐by‐experience collaborated with the authors in developing and finalizing the interview schedule. A summary of the questions asked during the interview is shown in Figure 1.

Three questionnaires were sent to participants to provide further contextual information relating to symptom presentation of each participant (Patient Health Questionnaire 9 (PHQ‐9; Kroenke et al., 2001), Generalized Anxiety Disorder 7 (GAD‐7; Spitzer et al., 2006) & Obsessive‐Compulsive Inventory (OCI; Foa et al., 1998)). The descriptive categories for participants' PHQ‐9 and GAD‐7 scores are shown in Table 1, along with total scores and focus of obsessions from the OCI.

### Data analysis

Interviews were analysed using established IPA procedures (Smith et al., 2009; Smith & Nizza, 2021). In summary, transcripts were read several times, with initial reflections noted in the reflexive journal. Detailed, line‐by‐line descriptive coding and interpretative coding were conducted, and initial themes from each transcript collated in a spreadsheet. Superordinate themes were identified, starting with individual interviews and broadening to identify themes across cases. Mind maps were used to aid this process. A more interpretative account of the themes and the meaning of these was developed through a reflection of the research team on both the data and existing psychological knowledge.

### Quality control

An audit checklist was created for this study based on the requirements suggested by Akkerman et al. (2006) and Larkin and Thompson (2012). An audit trail was recorded by keeping all relevant documents, data, and analysis throughout the study. The Audit was conducted during regular meetings with the second and third authors and at the end of the analytic process by the third author.

### Reflexivity

In IPA, transparency and reflection on the experiences, views, and preconceptions of the researchers is essential, as well as thinking about how these influences may impact on their interpretations of the data and its themes (Biggerstaff & Thompson, 2008). The first author kept a reflexive log throughout the research process (see File S2 for an excerpt), noting down emerging thoughts, reflections, and any links with prior knowledge which came up, and discussing these with the other authors.

## RESULTS

The analysis of the data produced six superordinate themes, presented below (see Table 2 for summary). Quotes from participants are included to illustrate each theme. The contributions of participants to each theme are given in Table 3. Material that has been omitted from quotes is indicated by ellipses ‘…’, and words added to enhance clarity and understanding for the reader are in square brackets ‘[]’.

**TABLE 2:** Superordinate themes and subthemes.

Superordinate themes	Subthemes
Submersion in intense and multifaceted imagery	
Overwhelming, uncontrollable imagery	Battling with imagery
	Intentional use of imagery
Imagery is explosive and expansive	From image to catastrophe at lightning speed
	Negative self‐judgement
	Hypervigilance to possible threats
Imagery involves past memories and future fears	Snapshots of trauma drawn in to imagery
	Susceptible to image collection
	Responsibility for imagined future harm
People respond to imagery as if it is real	An inherently emotional experience
	Imagery prompts compulsions and safety behaviours
	Irrational responses and self‐criticism
Therapy shifts imagery	

**TABLE 3:** Participant contributions to themes and subthemes.

Superordinate and subthemes	Participants							
	Dan	Hattie	Rachel	Amiera	Carys	Saskia	Natalie	Bridget
Submersion in intense and multifaceted imagery	✓	✓	✓	✓	✓	✓	✓	✓
Overwhelming, uncontrollable imagery	✓	✓	✓	✓	✓	✓	✓	✓
Battling with imagery	✓	✓	✓	✓		✓	✓	✓
Intentional use of imagery	✓	✓	✓	✓		✓		
Imagery is explosive and expansive								
From image to catastrophe at lightning speed	✓				✓	✓		✓
Negative self‐judgements	✓	✓	✓	✓	✓	✓	✓	
Hypervigilance to possible threats	✓	✓		✓		✓	✓	✓
Imagery involves past memories and future fears								
Snapshots of trauma drawn into imagery	✓	✓	✓		✓	✓		✓
Susceptible to image collection		✓	✓	✓	✓	✓	✓	✓
Responsibility for imagined future harm	✓	✓	✓	✓	✓	✓	✓	✓
People respond to imagery as if it is real								
An inherently emotional experience	✓	✓	✓	✓	✓	✓	✓	✓
Imagery prompts compulsions and safety behaviours	✓	✓	✓	✓	✓	✓	✓	✓
Irrational responses and self‐criticism	✓	✓						✓
Therapy shifts imagery	✓	✓	✓		✓	✓		

Unsurprisingly, the concept of responsibility was evident across all the themes, as would be expected (e.g. Salkovskis, 1999). For all participants, it was clear that living with OCD could be distressing, and had a large impact on many aspects of their lives.

“OCD just robs so much joy…” (Bridget).

The purpose of the current study was to investigate people's experiences of imagery, and so the following themes will explore this in more depth.

### Theme 1: submersion in intense, multifaceted imagery

Every person interviewed shared that they experienced imagery in a multisensory way, describing visual, auditory, olfactory, and physiological characteristics of their imagery in varying combinations. All participants described visual images; the content of these visual images varied depending on the person's specific obsessions, but they were always unpleasant and intense:

…so I get bodies, images of bodies and murdered people… (Bridget)

…that person who I feel like I might have offended… them, sort of, not smiling at me or not wanting to engage with me… (Rachel)

I can see the kitchen, and then just fire and smoke everywhere… (Saskia)

As well as purely visual images, participants also described imagery as having an auditory aspect, generally accompanying the visual images and appropriate to the scenario within the image, adding to its multifaceted nature. Vividness of auditory imagery varied from more muted accompaniments of visual images to extremely vivid, purely auditory imagery, as described by Dan:

I can almost hear the dynamic [of the water leaking], and not just like a ‘sss’, like a ‘SSSHHHH’ sound… the whole dynamic of it and the timbre of it, and I'll get that in detail…

Two participants described thje scent as being part of some of their images. For one, scent was present if the image was similar to a memory that had an olfactory aspect at the time, whilst for another, scent was an integral component of his whole experience of imagery:

…I could smell the sort of environment… if I'd noticed anything at the time… (Natalie)

I'm picturing everything, I'm seeing it, I'm smelling it… (Dan)

Imagery also had tactile and proprioceptive qualities for three of the participants. These qualities were consistent with the feared scenario and experiencing them preceded related compulsions. These feelings also seemed to be similar to a felt metaphor for a sense of threat:

…I feel that impact of it, how it would feel, the car hitting into it, and how my body would react to that… (Carys)

I feel like… sticky, grimy, yucky, all those types of things… (Amiera)

…I feel very small… I feel like there's something over my head about to drop… (Saskia)

Participants used expressive, evocative language to describe their experiences, demonstrating the vividness, detail, and intensity of their imagery, and indicating that this was a distinct and memorable feature of the imagery. These descriptions tended to embody the unpleasantness of the image and illustrate the submersion of the participant within the imagery itself. Participants' accounts were sometimes so detailed and vivid that the reader cannot help but conjure up the image in their own mind:

Like the most horrible green you could think of. Not like a nice emerald green. I'd describe it as like bogey green, like not very nice. Like the kind of green you'd associate with sickness, with a little bit of, tip of like brown‐ish, I guess… (Hattie)

…my brother made a fire… and all I could see was his body on the floor in flames… and me trying to, like, put the flames out. Like, just, vivid… (Carys)

Participants tended to report that their OCD and non‐OCD imagery was similar in the high level of vividness and detail, although people's experiences did vary to some degree. The major difference between OCD and non‐OCD imagery, however, was that non‐OCD imagery was not unpleasant, and focused more on everyday things. Some participants experienced non‐OCD imagery as actively pleasant, describing planning for events such as holidays or what to wear on weekend occasions. Others found it to be neither pleasant nor unpleasant; just neutral. This suggests a clear distinction between OCD imagery and non‐OCD imagery in terms of pleasantness.

…if I was going out somewhere, or planning, like, a holiday or going somewhere, it would just be nice thoughts… (Amiera)

… just like images of like what is going on, and like things that I might do later, so like what I might have for tea, I can picture what is in my fridge and things like that… (Hattie)

In summary, all sensory modalities were represented when participants spoke about their imagery. The vividness and detail of the images people experienced were important characteristics and seemed to emphasize the unpleasantness and intensity of the images.

### Theme 2: overwhelming, uncontrollable imagery

Participants spoke about experiencing OCD imagery as out of their control, and imagery occurring spontaneously without them voluntarily conjuring it up, often feeling overwhelming. Imagery was described as appearing immediately after hearing only part of a trigger, or a novel image appearing unexpectedly during a regular compulsion. Imagery was also hard to stop once it had started, with people not feeling in control of this.

I drive for 40 minutes to work, there and back, and it's just images of me crashing… (Carys)

I've only got to hear a syllable [of a trigger word]… It's an automatic response. I'm not thinking “oh I don't like that word, I think I'll worry about it”, it's an instant physiological reaction… (Bridget)

Yeah, it literally caught me off guard. It just happened… (Saskia)

### Battling with imagery

Several participants described feeling out of control of what their brain was doing, and the intensity of imagery was experienced almost as if the imagery itself had its own autonomy. As a consequence, some participants saw themselves almost as if in a “battle” with it. Participants seemed to experience this separation from being in conscious control of their minds as understandably unpleasant and frustrating:

…my brain will turn on when it wants to almost fool me, trick me into thinking there's something wrong… (Dan)

…OK, I'm done with it [the intrusive image], my brain's like ‘but are you really done with it?’ and I'm like oh, for goodness' sake, give me a break, you know… (Rachel)

For Amiera, this lack of control permeated into imagery being present within her dreams, and turning them into incredibly unpleasant experiences. No other participants mentioned dreams, but this was not specifically asked about in the interview schedule so it may be that it is more widespread an experience than reported here.

Like, my mind automatically thinks of contamination… it just turns the dream into a nightmare…

This battle did not seem to arise with non‐OCD imagery, with participants instead feeling less like the imagery was in charge, and more like they were:

I guess it's [non‐OCD imagery] different in that it's on my terms, rather than OCD's terms… I feel more in control of that… (Rachel)

#### Intentional use of imagery

Two participants described using imagery intentionally to mentally review events. This appeared to involve replaying or retelling intrusive images as a form of checking, possibly even as a ‘convenient’ by‐product of the imagery occurring:

I just remember having the image of the book on the desk and I was like, ‘Oh, have I left everything in my room?’ So I imagined everything left in my room. So that flashed in, and I was like ‘OK, yeah, you did that right. Did you do everything else right?’ (Saskia)

The experience of intentionally using non‐OCD imagery was quite different; participants described intentionally conjuring up non‐OCD imagery as a helpful strategy, either for work, hobbies, or relaxation. Everyone who spoke about these experiences was clear that whereas OCD imagery was spontaneous and uncontrollable, even when they then used it intentionally, their experiences of non‐OCD imagery included a feeling of control.

I made a spreadsheet today [for work]… and I visualised it in my head before it was there… (Dan)

…when I can't sleep… I imagine Christmas day, and waking up, and I plan the whole day in my head… (Saskia)

Overall, participants described feeling out of control of their OCD imagery, as if the imagery itself was in charge, and they were overwhelmed by this experience. Fewer people used imagery voluntarily as a form of checking, but more participants described using non‐OCD imagery for beneficial reasons, and this was a distinct experience, with them feeling more in control.

### Theme 3: imagery is explosive and expansive

This superordinate theme explores the common topic for participants that imagery was not a static experience; it developed into more elaborate, negative content.

#### From image to catastrophe at lightning speed

Participants described the content of their imagery rapidly progressing from the initial intrusive image to the image of a catastrophic feared future outcome. This generally had several steps of elaboration which happened quickly and without conscious effort. Participants' descriptions of the way in which imagery is rapidly elaborated also suggested a level of responsibility for the feared future event, and an interaction between thoughts and imagery which exacerbated this expansion.

…it probably does make me just go from, OK, blow‐out, accident, death (Dan)

I won't be able to use them [art supplies seen as contaminated] because if somebody bought that painting, it would contaminate them, make them ill, and kill them… (Bridget)

…if I meet up with my nieces and nephews, I imagine that I am contaminated and that I'm going to contaminate them and make them ill, and make them really ill… (Carys)

…it's when I'm checking the handle, and if I try to leave and it still doesn't feel right, it will pop in then, and it will be like ‘no, you need to check again, because that's [a break‐in] going to happen’, the re‐imagery… (Saskia)

#### Negative self‐judgement

Several participants spoke about how the imagery they experienced was elaborated to the extent that it suggested a deep, negative, meaning about themselves as people, and that they possessed undesirable traits which were opposed to their own values. For one participant, Amiera, imagery linked with her religion, which heightened the negative meaning she attached to herself because of the imagery, and built on the sense of imagery contravening moral rules. Rachel described a tension between logically knowing the imagery is opposite to her morals and values, but feeling like she may be a bad person for having the images.

It makes me really nervous that I'm a bad person, or that I'm racist… (Natalie)

…I try not to associate it with my personality, but you know there is always that ‘what if I actually feel like this and I actually want to offend this person’, even though it's like the furthest thing from what I want to do… (Rachel)

Rachel also described imagery as a ‘*double‐edged sword’* as she felt that the distress her images caused her had positive connotations for herself.

…sometimes I worry about getting over the images and the compulsions because I think that's going to make me less of a conscientious person or less of a kind person in other people's eyes… (Rachel)

This disparity between these two interpretations of the same imagery illustrates the complex and nuanced nature of people's experiences and highlights that it can be tricky for them to make sense of it themselves.

#### Hypervigilance to possible threats

Participants described paying close attention to their environment to be on the lookout for potential triggers or threats. For some people, this hypervigilance meant that common items they would see on an everyday basis were elaborated upon and interpreted as threats, feeding into the cycle of worsening imagery.

…so a lot of the time I'd walk around with a torch… my phone torch, especially if it's night, because I can't see, and I walk at a slow pace… (Amiera)

…if I saw any glass, or… any sharp pointy things, [they] were syringes and needles. So on the pavement, it could be a cotton bud… or anything long… or any glass, to me that becomes a syringe… (Bridget)

The experience of hypervigilance and OCD imagery being elaborated upon was contrasted with participants' experiences of non‐OCD imagery, which was described as more transient and fleeting:

I would say the image just passes. It just kind of like–as if it comes in one ear and goes out the other… (Hattie)

…like silly things like ‘what am I going to wear this weekend?’ and then I'll think about my wardrobe and then I'll go ‘oh yeah, OK’, and I'll move on… (Carys)

This theme highlighted that the content of imagery rapidly develops and changes throughout people's experience of it, holding a deep negative meaning about the self, with high levels of attention paid to the environment around them in case of potential triggers or threats.

### Theme 4: imagery involves past memories and future fears

Participants described their OCD imagery as being linked with memories as well as feared future harm.

#### Snapshots of trauma drawn in to imagery

Traumatic experiences described by participants varied from what psychological literature would call “Big‐T traumas” (James & MacKinnon, 2013), such as the death of a family member or abusive exposure treatment, to “small‐t traumas” (James & MacKinnon, 2013) which can have a cumulative effect, such as family members being unwell or a neighbour being burgled. Participants' descriptions illustrated imagery developing to include snapshots or elements of these traumatic experiences, either as a direct replication of the events, or as imaginary scenarios related to them.

I lost my cousin in a car accident… (Dan)

…barbaric flooding treatment…which the psychologists have acknowledged was abuse… (Bridget)

…my stepdad had Covid, and he got it really, really bad… (Saskia)

…I remember when I was 14 or 15… two houses up from us, they were robbed… (Saskia)

…similar to the hospital situations I've been in before, like exactly the same, like literally exactly the same… (Hattie)

…it is sort of lifted from an experience or an image that I know, but applied to me… (Rachel)

#### Susceptible to image collection

Some participants described aspects of their OCD imagery as being influenced by external sources, such as memories of things they have seen on television or in films. Often these additions were salient to the underlying sense of threat from their existing imagery, and integrated into their imagery experiences, even when the memories were from impersonal, more passive events.

It's like, you know like when you watch the criminal programmes and… there's like a handprint on it… it's like black… (Hattie)

…it's never happened to me so it's just something that I've probably seen on TV and stuff… (Carys)

I would say a lot of the time they're green because that's what I see from the film, Gremlins… (Amiera)

…it's just an absolute mess, as you see in films, it's just everywhere… (Saskia)

#### Responsibility for imagined future harm

In addition to OCD imagery being related to memories, the majority of participants described their imagery as having a focus on feared future harm. Participants described specific feared future events in which harm or upset would come to themselves or others, as well as more abstract imagery of future threats. Responsibility was a key thread running through participants' descriptions; they felt that what the imagery was depicting would be their fault.

…the visualisation of coming home to see it, and that was what was making me anxious… (Dan)

…because your mind has shown you doing it [stabbing someone], you're like, thinking ‘oh well, I've got to do this to stop that’, and if I don't do that, then I'm gonna do it… (Hattie)

What if I've picked up a virus from them [unwell children at work] and then I'll make them [young relatives] ill? And then I can see them like being poorly in bed… (Carys)

…the monster, it's something that I wouldn't want to be around, I have to run away from, then it's threatening for me, for what is to come… (Amiera)

Overall, this theme indicated that participants' OCD imagery drew influence from memories, both traumatic and more general, and that imagery of, and responsibility for, future harm was a consistent experience across participants.

### Theme 5: people respond to imagery as if it is real

A theme that was common across participants was that they responded to their OCD imagery as if it were real, in terms of emotional responses, behavioural responses, and obsessive or compulsive responses, including safety behaviours.

#### An inherently emotional experience

Every participant spoke about the emotional reaction to imagery, showing that it was a key feature of their experiences. For many, anxiety or panic was the primary emotional response, but participants also reported that their imagery elicited low mood. Imagery could also lead to feelings of frustration, and concentration was impaired for some participants as a result of the emotional responses to imagery.

I think it's just that feeling of anxiety really… it can sort of affect my concentration (Rachel)

…I get a feeling of dread, or I get really anxious… (Natalie)

…that felt really frustrating for me and I was very anxious… (Amiera)

It definitely doesn't help my mood. Yeah, it usually kind of leaves me feeling a bit shit… (Natalie)

#### Imagery prompts compulsions and safety Behaviours

All of the participants talked about having behavioural responses to their OCD imagery. Often imagery would prompt actions which would be described as compulsions or safety behaviours, such as repeated checking, reassurance‐seeking, and neutralizing. One participant, Dan, shared that he had made a significant financial investment (buying a new car) in response to his imagery and as an attempt to prevent future imagery from occurring. Other participants' responses were on a smaller scale, but their behaviours were still impacted:

…it [imagery] does lead me to doing… reassurance‐seeking and a lot of checking… (Rachel)

…I've asked people serving me [in a supermarket] ‘oh is that a water bottle [as opposed to a feared item]? Can you convince me? Can you write down that that's a water bottle?’ (Bridget)

#### Irrational responses and self‐criticism

Some participants mentioned feeling that their responses to imagery were irrational or illogical, but that despite being aware of this, they could find it hard to hold on to the rational perspective. This could then lead to self‐criticism, and feeling that they were being ‘silly’ or ‘stupid’ for responding in such a way. Dan's descriptions of the dichotomy between his logical knowledge about what he was experiencing and the irrationality of the responses his imagery elicited illustrate how frustrating and upsetting this experience was, and how he was critical of himself for thinking and responding in such a manner:

…it's ridiculous, you know, when you think about it rationally, why? But that's how it is. (Dan)

…the worst thing about it is I know it doesn't need to be checked, I know it's a problem up here [gesturing to head] but I can't help it, and that's the worst thing about it… (Dan)

By contrast with their responses to OCD imagery, participants tended not to respond to non‐OCD imagery, and the self‐critical reaction was also not present. Consistent with their description of non‐OCD imagery as more fleeting than OCD imagery in Theme 3, participants did not report any urges to act or respond to their non‐OCD imagery.

You wouldn't get like the panic or the guilt, or like the stress. Like there's no stress behind it, or like ‘Oh why have I thought that? I must do this to get rid of that image’–there's none of that… (Hattie).

This theme highlighted that OCD imagery triggered powerful emotions, which drove behavioural responses to the images. There was also a secondary, self‐critical analysis of this response as ‘ridiculous’ and irrational.

### Theme 6: therapy shifts imagery

Therapy was helpful for some participants in shifting their understanding of imagery or improving their imagery. Saskia had not realized that her experiences could be described as imagery until she spoke about them with her psychologist; this then helped her to understand imagery as part of her OCD. Other participants spoke about how doing behavioural experiments as part of their CBT had actively shifted the content and impact of their imagery, meaning they were more able to do everyday things such as go outside or have drinks made for them.

I didn't think it [imagery] meant a lot until [psychologist] had said this to me, and then I was like ‘Oh, OK, actually I do get quite a lot’… (Saskia)

It probably helps actually because for the last week I've asked someone to make me a cup of tea every day… and the week before I was proper panicking like ‘I'm going to get ill in the next 12 h… get a stomach bug, vomiting bug’. Whereas towards the end of this week… I wasn't spiralling… (Carys)

Also captured within this theme is that some participants reported that the research interviews themselves, with discussion around imagery and what it means and involves, was helpful in bringing imagery to their awareness and shifting their relationship to it, despite this not being a clinical intervention.

…an observation from reading your leaflet and the correspondence we've had is that I never really thought about it as sort of imagery… I've always just thought of it as intrusive thoughts… but in a way… it's absolutely imagery as you've sort of gone through that, and it's just a helpful perspective… a thought is just a thought and an image is just an image, so I think it will also just help with the separation a little bit more, so definitely have a positive way of thinking of it… (Rachel)

…I picked up on things talking today to you that maybe I've not noticed before… (Dan)

In summary, this theme indicated that therapy that is not specifically imagery‐related can still have a positive impact on people's experience of imagery. It also suggests that simply speaking to someone about their experiences of imagery was a helpful experience for participants.

## DISCUSSION

The current study found that participants described their OCD images as vivid and detailed, primarily visual but incorporating other sensory modalities, and often distressing, consistent with previous research (Rachman, 2007; Speckens et al., 2007). The theme of OCD images being elaborated upon, however, is at odds with Rachman's (2007) claim that “…images change very little from occasion to occasion. They display remarkable stability and consistency…” (p.405), and indicates that previous conceptualizations may not have captured people's full experiences of OCD imagery.

All participants shared that their imagery was based on past memories, which could be traumatic and/or more everyday memories. The findings of the current study are consistent with Speckens et al. (2007), who found that images were memories of, or associated with, earlier negative events for two‐thirds of participants, and also align with Miller and Brock's (2017) meta‐analytic findings of a significant relationship between past trauma and OCD symptoms. In slight contrast, Lipton et al. (2010) found that only 15% of OCD participants said their image was directly connected to a memory, and 55% said it was associated with an earlier event, leading the authors to conclude that imagery in OCD is “derived more from fantasy and imagination” (p. 821). Taking all of this together, there seems to be a consistent finding across studies, including the current study, that OCD imagery is often associated with earlier memories or events, but can also include elements of elaboration and imagination, which is corroborated by the findings from the current study.

For all the people who participated in this study, it was evidenced that both memories of past events and fears about future incidents were implicated in their experiences of imagery. Consequently, this supports the Salkovskis (1999) cognitive model of OCD, which states that obsessional intrusions often focus on future fears, and Rachman's (2007) description of OCD imagery encompassing past events but also includes anticipatory future‐focused images. This finding also suggests that solely addressing past traumas in the treatment of OCD is unlikely to be sufficient, and there is a need to integrate this with examining feared future events, for example, as in the CBT treatment model for OCD (Bream et al., 2017).

The findings from this study could inform many aspects of clinical practice with people who have OCD. It is clear that imagery is a very significant part of people's experiences of OCD; images are very vivid and have a big impact on people's everyday lives, and this needs to be held in mind by clinicians throughout the clinical cycle. Assessment will be a key point at which clinicians should be explicitly asking about imagery, as people may then feel empowered to share their experiences of imagery which may have otherwise been missed in a standard assessment process.

The findings from the current study make suggestions as to how imagery could be integrated into the formulation. Thinking about the commonly used CBT model of OCD (Salkovskis, 1999), the findings of this study suggest that imagery is present in all parts of this process. This will be important for therapists to hold in mind when formulating using this model, for example identifying intrusive images as well as thoughts, considering whether images act as triggers for obsessions and compulsions and whether the person may intentionally use imagery as a response (i.e. replaying images as a form of checking). Imagery of past experiences may also contribute to responsibility beliefs and intolerance of uncertainty, which are thought to be present amongst many people with OCD (O'Leary et al., 2009; Pinciotti et al., 2021). To further inform formulation, people with OCD could be encouraged by their therapist to create and engage with an image of their feared stimuli, to help them think about how they think, feel, and behave when exposed to these stimuli. This could also act as a first step towards behavioural experiments.

People responding to OCD imagery as if it is real will be a particularly important concept to consider when working therapeutically with people with OCD. This finding suggests that responses to the presentation of feared stimuli through approaches such as virtual reality (VR), a technique which has already been found to prompt anxiety and an increase in OCD symptoms (Dehghan et al., 2022), may be reflective of the responses to in vivo feared stimuli, and so could be a more practical way of carrying out behavioural experiments.

OCD imagery conveying meaning about the self was a consistent subtheme across the majority of participants, with strong negative connotations; this aligns with findings from Speckens et al. (2007) and Lipton et al. (2010) around the inferences people drew from their imagery that the self is “bad” or “dangerous”. This finding is also consistent with and provides support for the role of the feared self as an important construct within conceptualizations of OCD (e.g Aardema & Wong, 2020). Based on this, there are clinical implications for the treatment of OCD, for example, targeting this feared self, or negative self‐concept, with the use of inference‐based CBT (Aardema et al., 2022). It was not evident whether participants in the current study experienced this negative self‐concept as imagery, and so further studies to investigate this would add depth and clarity to the concept.

The findings of the current study indicate that alterations in imagery or responses to imagery link with reduced OCD symptoms and impact; it could therefore be helpful to consider asking about any changes or reductions in imagery, or responses to imagery, as part of the evaluation of the therapeutic process.

Across themes, the current study found that OCD and non‐OCD imagery were experienced very differently by participants; notably in intensity, the emotions they elicit, the self‐appraisal following imagery, and the level of control over the imagery. Despite being phenomenologically distinct, all participants experienced both non‐OCD and OCD imagery, positing some interesting questions regarding the possible mechanisms by which imagery “becomes” OCD‐related, or vice versa. Imagery mechanisms in clinical populations is an under‐studied area (O'Shea & Moran, 2019), and so future research could investigate the processes behind these distinct categories of imagery, if there are differences in susceptibility to experiencing imagery and responding to it, and how these may contribute to the development and treatment of OCD.

There were some limitations in this study. Participants were predominantly female, white and in their mid‐to‐late twenties. This is not reflective of the clinical population of people with OCD and may mean that the experiences shared by many of the participants in this study may differ from those of people who are of different genders, ethnicities, and ages, and means the voices of these people are underrepresented in this study, and that the findings may lack transferability–a risk in all qualitative research (Spencer & Ritchie, 2012). Recruitment from NHS services may have meant that participants were also experiencing more severe or intense OCD symptoms, which may not accurately reflect the range of experiences of people with OCD. However, asking participants to complete the Obsessive‐Compulsive Inventory (Foa et al., 1998) was a helpful measure to add contextual information about symptom severity, and showed that there was a range of severity between participants. It will be important to capture the experiences of people from a wide variety of backgrounds, to see where their experiences of imagery are similar and different to those captured here. One potential way to increase diversity and representation in future studies would be to recruit from outside of NHS services. The current study advertised via the charity OCD Action, however, recruitment was unsuccessful; a possible reason behind this may have been that the online advert was not easily accessible to people casually browsing the website. Future research may wish to use avenues with wider reaches, such as social media, to encourage participation from a broader, more diverse, range of people.

It is important to acknowledge the perspectives brought by the authors as clinicians working within a framework based largely upon CBT, and with extensive knowledge and experience of this model. Whilst this may have aided a nuanced understanding as to how the phenomenon of OCD imagery mapped onto existing conceptualizations and knowledge, it also means that there may have been a risk of novel material being missed. That being said, our careful approach to considering reflexivity and the detailed analytical approach means that this risk was small. The involvement of an expert‐by‐experience was a strength of this study, particularly in the development of the interview schedule. Future research would benefit from building on this and working towards co‐production of research, including co‐analysis, which would add novel perspectives and richness to the analysis.

## CONCLUSION

This study aimed to investigate people's experiences of mental imagery in OCD through semi‐structured interviews and Interpretative Phenomenological Analysis. The analysis revealed six superordinate themes: Submersion in intense and multifaceted imagery; Overwhelming, uncontrollable imagery; Imagery is explosive and expansive; imagery involves past memories and future fears; People respond to imagery as if it is real; Therapy shifts imagery. Clearly, this adds to the existing conceptualization of OCD that states that imagery is a key experiential aspect of the condition, however, our work uniquely demonstrates that this imagery contributes to the overall experience of people seeking treatment for OCD. It is already recognized that such imagery must be considered when formulating treatment plans (e.g. Hackmann et al., 2011; Stopa, 2021), however, clinicians do need to ensure that they are assessing and seeking to understand individualized accounts of the role imagery plays in the maintenance of OCD, and the distress experienced by those living with the condition.

## AUTHOR CONTRIBUTIONS

**Hannah E. F. Wedge:** Conceptualization; data curation; formal analysis; investigation; methodology; project administration; resources; validation; visualization; writing – original draft; writing – review and editing. **Louise Waddington:** Conceptualization; formal analysis; methodology; supervision; validation; writing – original draft; writing – review and editing. **Andrew R. Thompson:** Conceptualization; formal analysis; methodology; supervision; validation; writing – original draft; writing – review and editing.

## FUNDING INFORMATION

This research did not receive any specific grant from funding agencies in the public, commercial, or not‐for‐profit sectors.

## CONFLICT OF INTEREST STATEMENT

There are no conflicts of interest to disclose.

## Supporting information


File S1.

File S2.

## Data Availability

The data are not publicly available due to privacy and ethical restrictions, as they contain information that could compromise the privacy of research participants.
