# Supplementary material for: Experiences of imagery in obsessive‐compulsive disorder: An interpretative phenomenological analysis
Source: Br J Clin Psychol. 2024 Dec 5;64(2):491–512. doi: 10.1111/bjc.12518 (PMC12057309; doi:10.1111/bjc.12518)
Supplement: Supplementary file 1 — File S1. [file BJC-64-491-s002.docx]

**Supplementary File 1**

Interview Schedule

**Introduction**

- Before we begin, are you in a quiet, private space where you will not be interrupted?
- Have you had time to read the information sheet? If not, we can go through it together now.
- If you are willing to take part in this study, please sign this consent form. You are under no obligation to do so. Do you have any questions?
- There will be three questionnaires for you to complete before the interview, if you haven’t brought them with you today. Please ask if you need help or explanations for any of these.
- You are able to ask me questions at any time during the interview.
- You can pause the interview for a break if you need or want to. You can also stop the interview completely at any time, for any reason.
- You may find that some of the things we are going to talk about today in relation to your OCD might be upsetting for you. Please let me know if you would like to pause or stop the interview.
- If you share something that makes me worried about your safety or the safety of other people, I have a duty of care to share that with the team responsible for your care.
- The final report I write may use some quotes from your interview. These will be anonymous and will not contain any details which could be used to identify you.

**Demographic Questions**

Before we begin the main interview, I’d like to get some background information which I will use anonymously when presenting the results. I’ll only need brief answers to these questions.

- What gender do you identify as?
- What are your preferred pronouns?  (How do you like to be referred to – he/she/they/another term?)
- What is your age?
- How would you describe your ethnicity?
- When were you diagnosed with OCD?
- Have you received any psychological treatment or therapy for your OCD, and if so, what?
- Do you have any other psychological conditions and/or diagnoses that you think it would be useful for me to be aware of? If yes, what?

**Interview Schedule**

We are now starting the interview itself. I would like to ask you about your experiences of imagery in relation to your OCD. Imagery has been described as like having a sensory experience without the physical stimulus or object. Although it is often visual, imagery can involve any of the five senses – sensations, sounds, smells and tastes, as well as images/pictures.

1. With this in mind, could you please bring to mind a **typical recent example** of imagery you experience to do with your OCD.
2. Can you **describe the imagery** to me in as much detail as you can?
   1. *P: Can you tell me if there were any other senses involved in this imagery?*
   2. *P: Can you walk me through what happened, starting just before the imagery came to mind?*
3. What impact does this imagery have on you?
   1. *P: Another way of putting this might be what effect does it have on you? Your mood, body, daily activities, etc.*
   2. *P: Could you tell me a bit more about if the imagery has any effect on what you think or do afterwards?*
4. Does this imagery hold any meaning for you?
   1. *P: Another way of thinking about this is whether you feel the imagery suggests anything about you, other people, the world around you, the future, etc.*
   2. *P: Does this meaning link to anything that has happened before or might happen in the future?*
5. Are there any other forms or types of imagery that you experience related to your OCD? If so, what is that/are they like?
   1. *P: Another way to think about this could be whether imagery makes up a large part of your experience of OCD, or if it is a smaller aspect of it.*
6. Do you experience imagery that is **not** related to your OCD? If you do, can you tell me about it?
   1. *P: How does this compare to when imagery is related to your OCD?*
7. Is there anything else you would like to add that we haven’t spoken about already?

**Debrief**

- Now that we have finished the interview, is there anything that you feel concerned or worried about?
- If, after you leave, you feel like you need some support around the things we have spoken about today, you can contact your clinician.
- You are also able to contact support services such as OCD-UK, Samaritans or Mind, should you feel that you need additional support (provide participants with list of services and contact information).
