# Supplementary material for: Experiences of imagery in obsessive‐compulsive disorder: An interpretative phenomenological analysis
Source: Br J Clin Psychol. 2024 Dec 5;64(2):491–512. doi: 10.1111/bjc.12518 (PMC12057309; doi:10.1111/bjc.12518)
Supplement: Supplementary file 2 — File S2. [file BJC-64-491-s001.docx]

**Supplementary File 2**

Excerpts from the analytic process


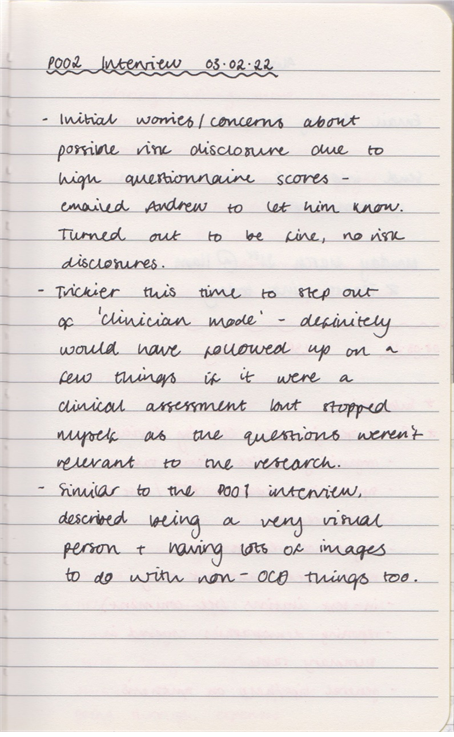
Excerpt from Reflexive Log:


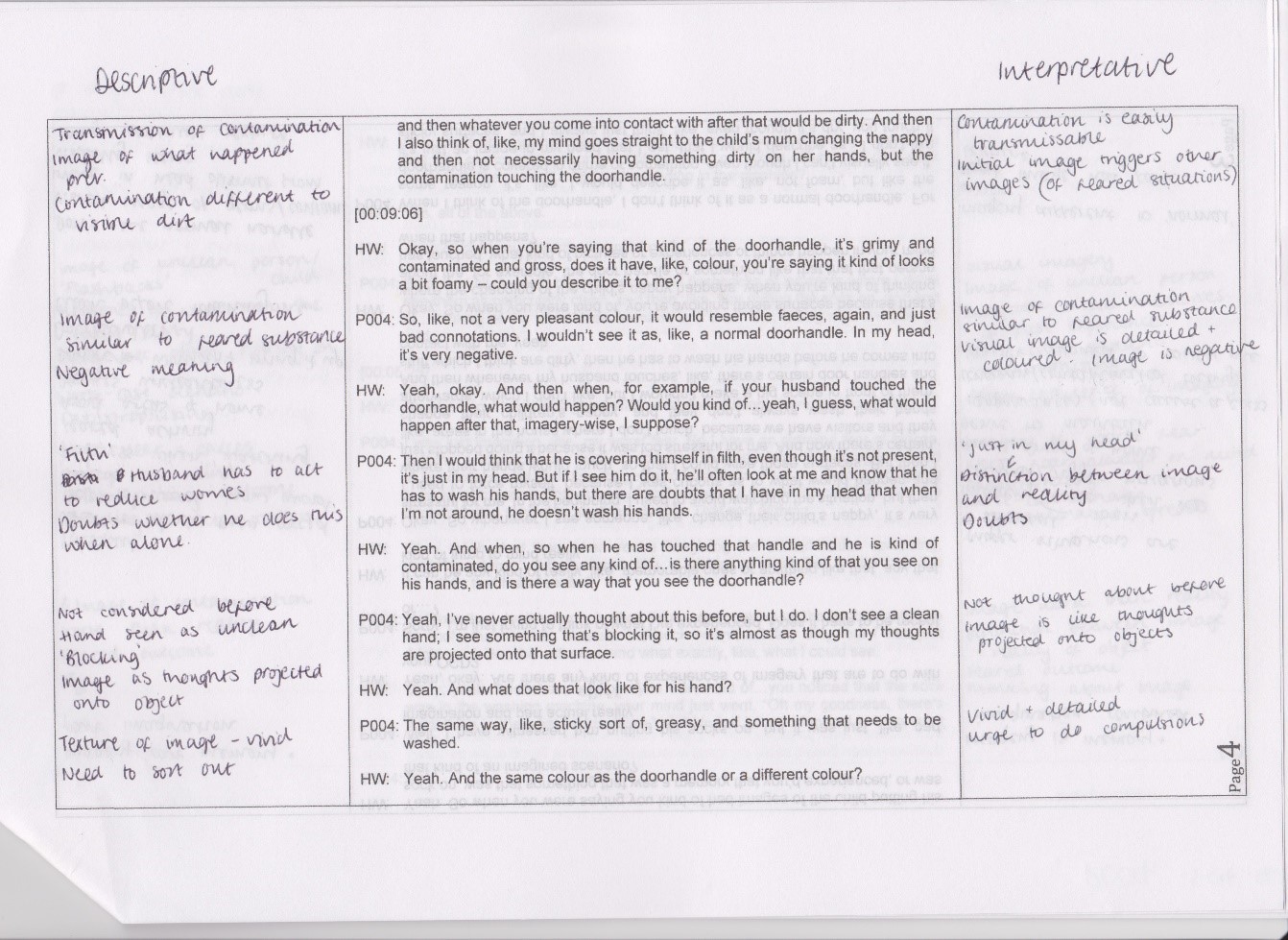
Excerpt from coding:


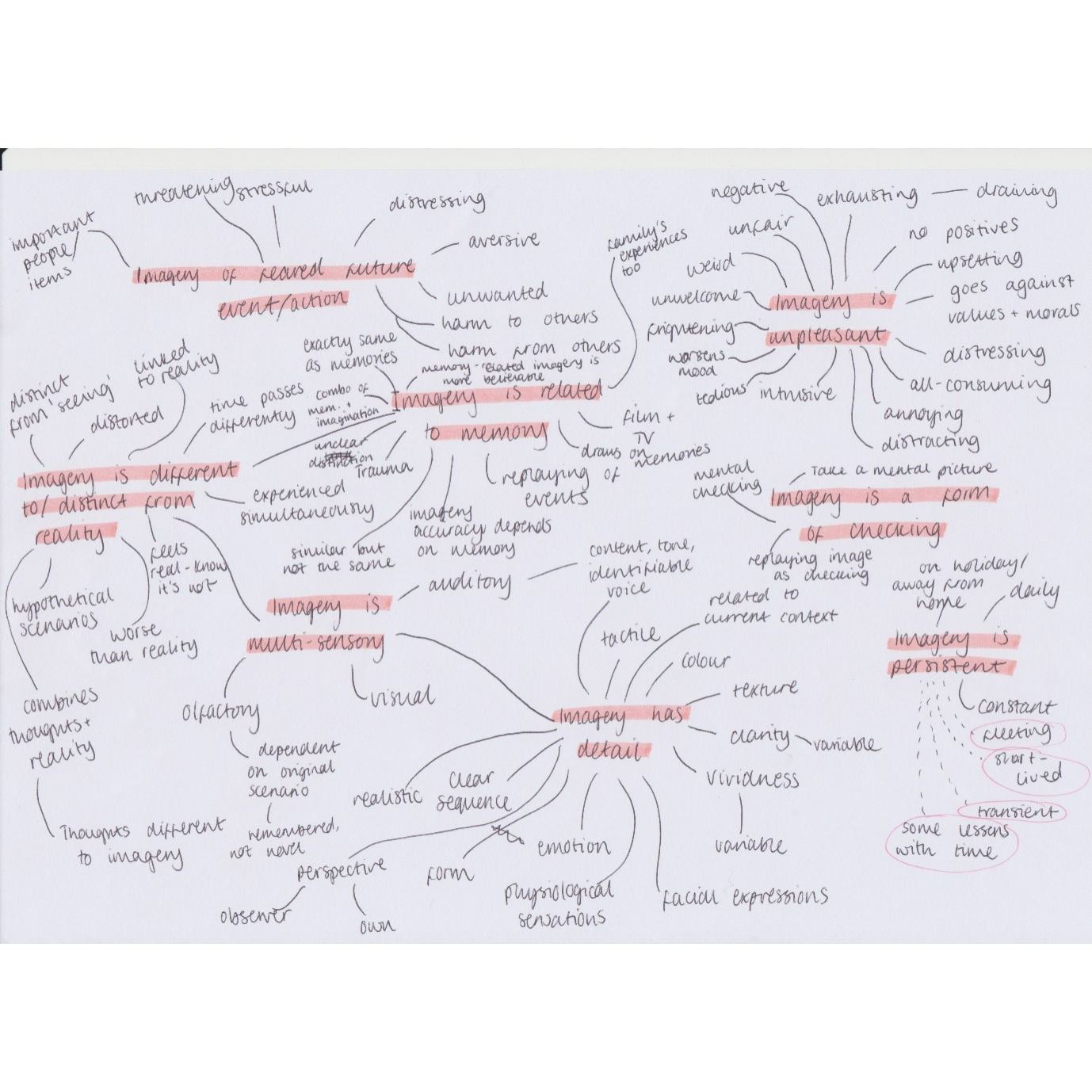
Example mind map:
